# The SMOOTH-Robot: A Modular, Interactive Service Robot

**DOI:** 10.3389/frobt.2021.645639

**Published:** 2021-10-05

**Authors:** Norbert Krüger, Kerstin Fischer, Poramate Manoonpong, Oskar Palinko, Leon Bodenhagen, Timo Baumann, Jens Kjærum, Ignacio Rano, Lakshadeep Naik, William Kristian Juel, Frederik Haarslev, Jevgeni Ignasov, Emanuela Marchetti, Rosalyn Melissa Langedijk, Avgi Kollakidou, Kasper Camillus Jeppesen, Conny Heidtmann, Lars Dalgaard

**Affiliations:** ^1^ The Maersk Mc-Kinney Moller Institute, University of Southern Denmark, Odense M, Denmark; ^2^ Institute for Design and Communication, University of Southern Denmark, Sønderborg, Denmark; ^3^ Department of Informatics, Universität Hamburg, Hamburg, Germany; ^4^ Dictus ApS, Brøndby, Denmark; ^5^ Department for the Study of Culture, University of Southern Denmark, Odense M, Denmark; ^6^ Danish Technological Institute, Odense M, Denmark

**Keywords:** mobile robots, service robots, human–robot interaction, socially aware navigation, proactive control

## Abstract

The SMOOTH-robot is a mobile robot that—due to its modularity—combines a relatively low price with the possibility to be used for a large variety of tasks in a wide range of domains. In this article, we demonstrate the potential of the SMOOTH-robot through three use cases, two of which were performed in elderly care homes. The robot is designed so that it can either make itself ready or be quickly changed by staff to perform different tasks. We carefully considered important design parameters such as the appearance, intended and unintended interactions with users, and the technical complexity, in order to achieve high acceptability and a sufficient degree of utilization of the robot. Three demonstrated use cases indicate that such a robot could contribute to an improved work environment, having the potential to free resources of care staff which could be allocated to actual care-giving tasks. Moreover, the SMOOTH-robot can be used in many other domains, as we will also exemplify in this article.

## 1 Introduction

**FIGURE 1:**
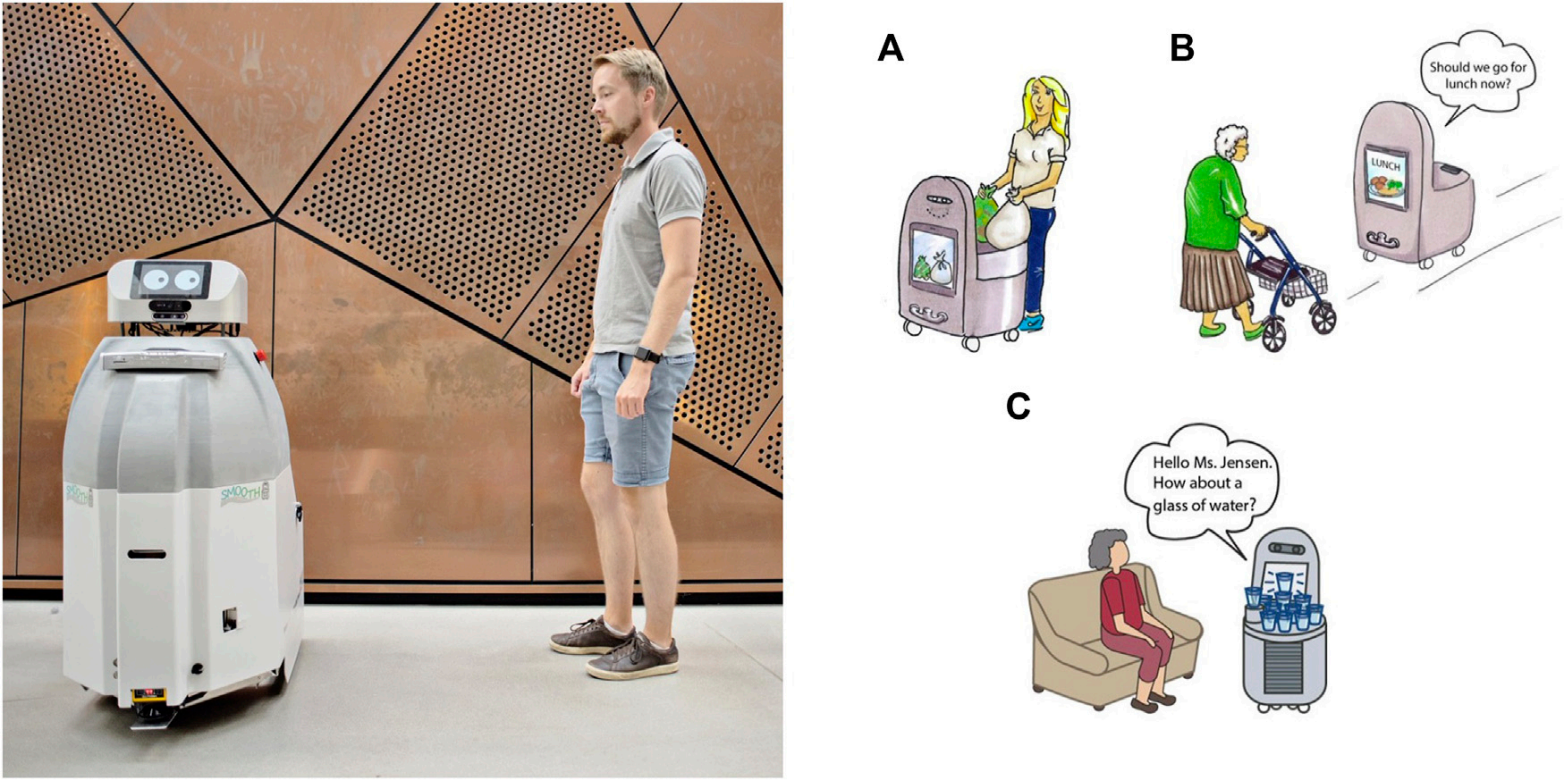
SMOOTH-robot **(A)** and the three use cases as outlined at the beginning of the project **(B)**; a) use case 1: garbage and laundry transport, b) use case 2: guiding, and c) use case 3: offering beverages. (Figure on the left provided by Maud Lervik/Nordisk Ministerråd. Figures on the right provided by the Danish Technological Institute.)

In this work, we introduce a novel service robot at a Technology Readiness Level (TRL) (HORIZON 2020, 2014) of 5–6 (see Figure 1A). The robot was originally designed to be applied in elderly care homes, but it possesses properties that make it applicable in many other domains also. Besides its design, basic functionality, and its application in elderly care homes and beyond, we will also describe the technical and business case-related challenges connected to entering the market with such a kind of robot and why our design choices could carve the path to success.

While the clear commercial success of care or social robots does not seem to have materialized yet (USA Today, 2018; Human Robot Interaction, 2019), the situation in mobile logistic robotics is different. Here, we see a market of significant size emerging, where companies such as MiR ^1^ and Aethon ^2^ sell thousands of robots a year that are able to operate in the vicinity of humans. These robots, however, in general, do not interact with humans but basically avoid them. Many logistic tasks could be solved better when at least some interaction capabilities would be present, for example, during the initialization and finalization (hand-over) of the transport of goods. Furthermore, even simple cues such as gazing toward the humans in robots surrounding or greeting them can significantly improve their degree of psychological comfort around the moving robot. Moreover, a lot of additional use cases could be tackled if fundamental HRI competences would be available on logistic robots.

The SMOOTH-project ^3^ aimed to design an assistive social robot that engages in (at least some basic) interaction with humans, which also leads to convincing business cases for end-users such that commercialization becomes feasible. The robot should be able to detect humans, navigate in a socially aware manner, understand human intentions using simple cues such as gaze detection, and communicate with humans *via* dialogue, gaze, and body orientation. Hence, it should interact with humans to a degree that is technically feasible today.

For that, we addressed four important factors in different ways than in the existing social robots:

Technical complexity: Although significant progress in navigation, computer vision, and speech recognition has been made over the last decade, there are still fundamental challenges, especially in the domain of smooth human–robot interaction, which not only requires the close-to-perfect functioning of all perception modules but also some knowledge about the likely reactions of humans to robot actions, that is, some kind of pro-activity. It is clear that with today’s technology, this can only be achieved for rather simple and repetitive tasks.

Degree of anthropomorphism: Anthropomorphism is a critical feature in human–robot interaction. On the one hand, humanlike features (such as gaze and gestures) allow a human to relate to a robot in terms of the cues he or she is used to. On the other hand, anthropomorphic features could lead to expectations that the robot (because of today’s technical limitations, see above) cannot fulfill (Chun and Knight, 2020). During the design phase of the SMOOTH-robot, we carefully adjusted the degree of applied anthropomorphism. Specifically, we use a robot gaze to initiate and modulate the interaction with the human partner, while due to its shape, our robot is still clearly identifiable as a machine. Hence, human expectations about the capabilities of the robot are adjusted to what a robot can actually deliver.

**FIGURE 2:**
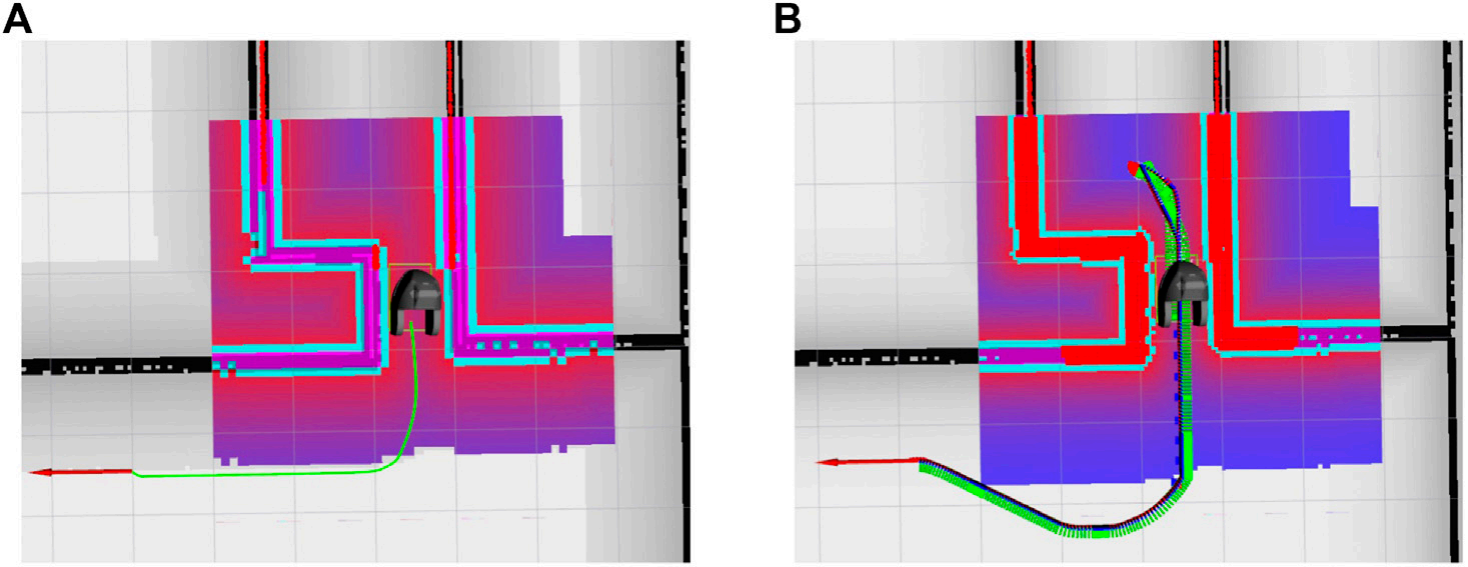
**(A)** Dijkstra planner wants the robot to turn on the spot in a tight corridor; **(B)** SBPL planner drives the robot to a location with enough space to turn around.

Wide range of applicability: The SMOOTH-robot is supposed to autonomously solve frequently occurring tasks that are usually conducted by care-givers or other staff. This implies that the robot needs to be able to transport, load, and offload items. In contrast to that of Pepper and Jibo, our robot body has a functional and not just a social purpose; in many use cases, it needs to carry and/or offer items in some meaningful way to humans (see Figure 2). The SMOOTH-robot can serve a large variety of use cases by loading and offloading modules that serve different purposes.

Affordability: During the design of the SMOOTH-robot, we aimed at a sales price in the range of €30–40,000. In addition to the rather affordable price, we reduced the running costs for applying the robot by enabling the very same robot to serve different purposes. The additional costs connected to a new use case are then restricted to purchasing a new module that can be produced much more cheaply than the robot itself. By that, idle times of the robot can also be reduced, which potentially increases the value generation for the end-users.

During the design process of the SMOOTH-robot, we carefully considered these four factors and arrived at an affordable robot which can be applied and can create value in a wide range of use cases. The SMOOTH-robot makes use of a moderate degree of anthropomorphism to initiate and modulate human–robot interaction. The tasks addressed are selected such that they can be solved at a complexity level that is feasible by today’s technology.

This article is structured as follows: after giving an overview of the state of the art in the field in Section 2, we present the design process of the SMOOTH-robot in Section 3. In Section 4, we describe the technical modules that are required to navigate the robot and allow for reasonable interactions with humans. Our experience with three applications of the SMOOTH-robot, two of which were tested in elderly care facilities, are presented in Section 5. A short summary of our work and consequences for future work are outlined in Section 6.

## 2 State of the Art

In the following overview of the state of the art, we first discuss social robots today (Section 2.1) and then the technologies that are required to realize human–robot interaction on mobile robots (Section 2.2). We also explain what technologies have been used and/or modified to be applicable on the SMOOTH-robot and in what respect we were able to go beyond the state of the art.

### 2.1 Social Robots

There are already a wide range of care robots in various shapes and functionalities on the market (see Bodenhagen et al. (2019) for a detailed review). To name just a few, the PARO-robot ^4^ has a seal-like appearance and is used, for example, to provide comfort to people suffering from dementia (Hung et al., 2019). It costs around €5,000. In Denmark, many municipalities bought PARO-robots, but the response has been very mixed and even slightly disappointing; many of these expensive PARO-robots are now lying unused on shelves, mainly because its application in elderly care homes has turned out to be too complex. As stated in the study by (Hung et al., 2019), “Most interventions conducted have been primarily researcher-focused. Future research should pay more attention to the clinical needs of the patient population.”

The Pepper robot is another example of a service-robot. It is an affordable small-scaled humanoid robot. It is made available in a leasing model of $360 a month, which accumulates to more than €10,000 over 3 years (The Robot report, 2016). Pepper has been applied in elderly care [see, for example (Tanioka, 2019)]; however, it is open to debate as to whether its use can be considered a success (Bloomberg, 2020). One main problem has been that the robot’s body is fixed and cannot be used to transport items, which might be decisive in addressing commercially relevant use cases.

A more expensive assistive robot is the Care-O-bot 4 developed by Fraunhofer IPA^5^, which comes in different shapes. A basic version without arms costs around €100,000, while a version with two arms costs more than €200,000. Care-O-bot 4 is manufactured by Mojin Robotics ^6^, a spinoff company from Fraunhofer IPA. The commercial version (without arms) has been used for guidance applications in retail stores and other application environments. The research version (with arms and object detection abilities) has been used for fetch-and-carry tasks and, lately, in the RoPHa project ^7^ to support users at the meal table, for example, by cutting food, sprinkling it, or offering single pieces in front of the user’s mouth. The robot’s high price makes it extra difficult to create an appealing return of investment.

The social robot Jibo ^8^—which has not been explicitly designed for elderly care—is one of a number of similar and rather simple nonmobile robots that were meant to be used in households. Jibo, which costs around €700, has been able to communicate *via* voice, rotate its body, attend to the person it is talking to, support its verbal expressions by gestures, and take pictures from a certain view point. Jibo Inc. needed to close down in 2018, and more than €50,000,000 (The Robot report, 2018) of investment was lost. As reasons for this failure, a couple of major problems have been discussed (The Robot report, 2018): 1) the high price compared to those of Amazon’s Alexa and Google Home, mainly due to the motors integrated in the embodiment, 2) technological issues that led to delays of deliveries and restriction of sales in the U.S. and Canada, and 3) similar competitive products designed and produced in other countries (e.g., in China). In the context of the last point, the necessity of being open about the product at an early stage, which is typical for crowd financing, turned out to be harmful.

### 2.2 Technologies for Human–Robot Interaction

Despite many research projects on human–robot interaction and significant progress in disciplines such as navigation, computer vision, speech processing, and dialogue design, mobile service robots that deeply interact with humans have not reached the market yet (Bodenhagen et al., 2019). Today’s mobile robots that are applied in industry and institutions such as hospitals generally avoid humans, that is, they do not engage with them. Robots that are able to interact with humans would improve the applications of robots in use already (e.g., by understanding human gestures to avoid potential collisions with the robot). It would also allow for a large variety of new applications (e.g., in use cases that require some information transfer to initialize or finalize the logistic operation, as, for example, in the beverage-serving use case).

This points to a general problem (Bodenhagen et al., 2019); while logistic and task-specific mobile robots such as cleaning robots are slowly entering the market, there still exist limits on technical feasibility and challenges that need to be overcome in areas where interaction with humans is required. In particular, the application of mobile robots with manipulators in public spaces is, in our view, unrealistic in the near future, due to safety reasons on the one hand, but also due to the cognitive prerequisites involved in the control of dexterous hands on the other (The Conversation, 2018). Furthermore, hardware limitations concerning a stable use of tactile information impose significant hurdles. While robot manipulation in constrained industrial environments with a high repetition of the very same or at least similar actions applied to similar objects under controlled illumination conditions is common, the variability of objects, users, and constellations in public spaces constitutes a major challenge that reduces the possible applications of such robots. In the SMOOTH-project, we therefore decided to not equip our robot with robot arms.

While the reliability of individual sensor modalities such as vision and speech has increased over the course of the last 20 years—in particular, through the application of deep neural networks—to a level where some rather specific tasks such as cancer diagnosis on medical images can be performed at a level matching and sometimes even exceeding human performance (Schmidhuber, 2014), the integration of such modules into satisfactory behaviors is still a challenge. Even the dream of fully autonomous cars—which appeared to be within close reach some years ago—seems now to be realistic only in a much more distant future (The Conversation, 2020).

A fundamental problem turned out to be the complexities of human interaction that require—besides perceptive skills—a high degree of cognitive and social skills, which even humans take many years to develop. The main reason for the difficulty of bringing robots out into human social spaces is the enormous complexity of human interaction, which is multimodal, incremental, and highly dynamic and has been optimized for efficiency over the course of thousands of years—optimized for human processing and human needs, of course. Humans use their body orientation, speed, gaze, mimics, gesture, and speech to coordinate their actions with those of others (e.g., Mondada (2018)), and due to incremental processing (Levelt, 1989), they can adjust to each other incredibly quickly and on several of these channels at the same time (Cameron et al., 2016; Jensen et al., 2020). Furthermore, humans share common ground based on previous joint experience, cultural knowledge, and human nature (Clark, 1996), which helps them orient themselves quickly as to what is going on. Thus, for humans, it is relatively easy to move in congested spaces or identify who would like to receive a glass of water and who would not because they can make use of a multitude of social signals to infer other people’s intentions and predict their behavior, which they interpret on the basis of extensive background knowledge.

**FIGURE 3:**
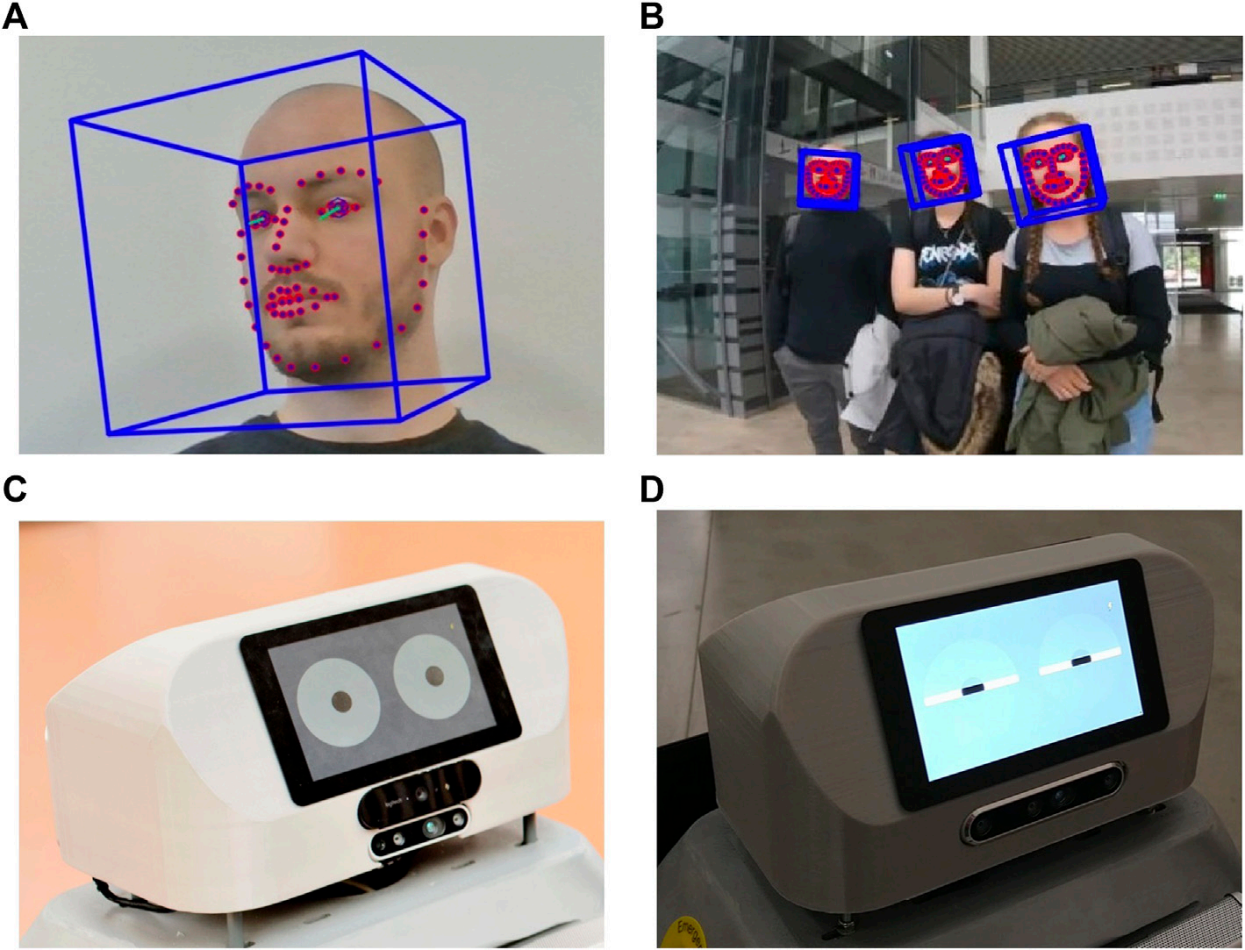
**(A)** Head pose and gaze estimate using OpenFace; **(B)** people’s head pose and gaze detected while interacting with SMOOTH; **(C)** SMOOTH’s head displaying its own gaze on the front screen; **(D)** SMOOTH blinking.

**FIGURE 4:**
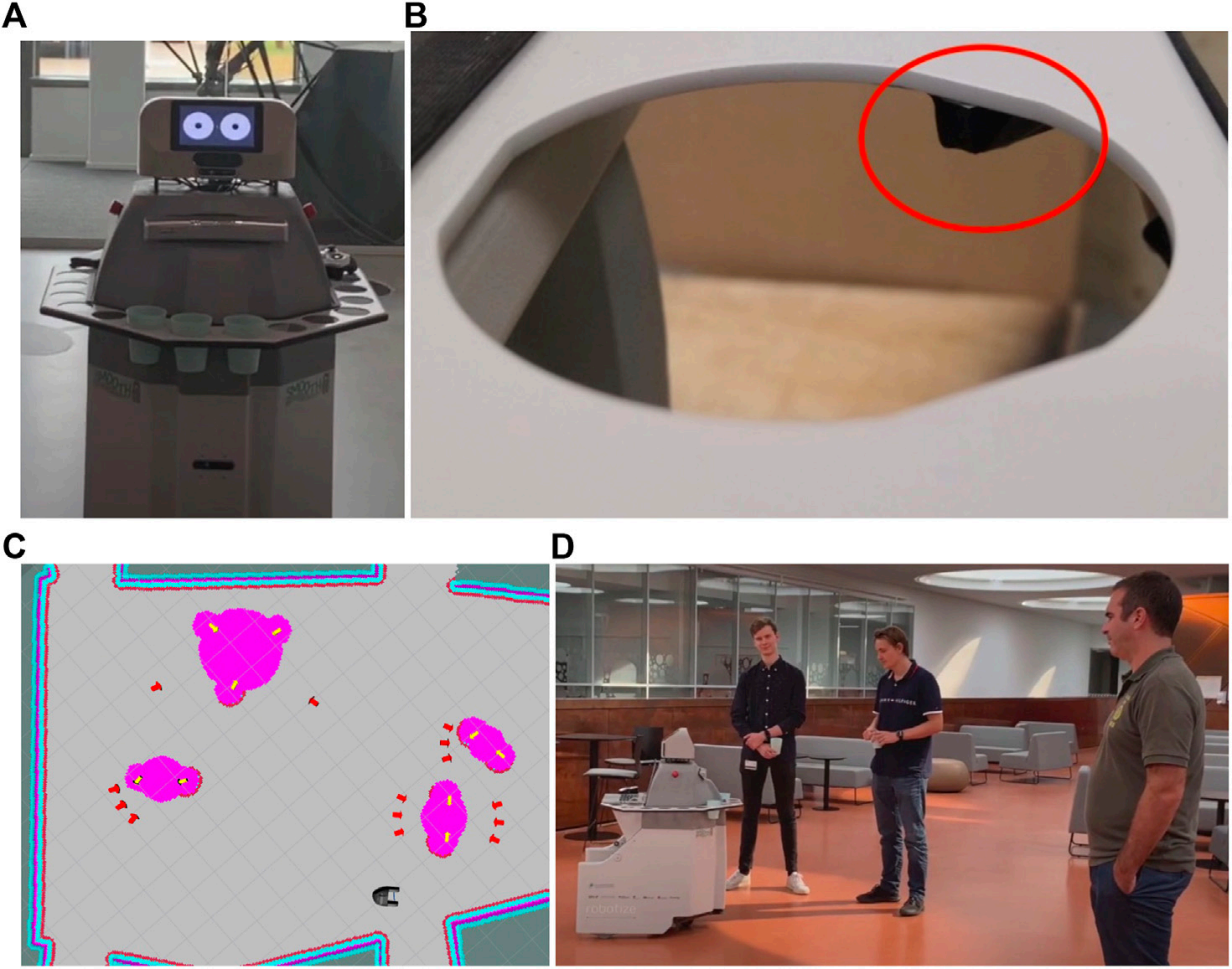
**(A)** SMOOTH with a tray for holding beverage cups; **(B)** tray sensor; **(C)** scenario 1 of beverage delivery use case—robot detects people (yellow arrows), identifies groups among them, adds costmaps around detected people and groups (pink color), and plans approach points for serving beverages (red arrows); **(D)** robot approaching groups of people in a sparse reception scenario.

The difficulty of interacting robots leaving lab environments and becoming commercial applications is especially relevant in the light of public expectations, which are often more driven by science fiction movies than the state of the art of today’s technology. One consequence is to balance the amount of anthropomorphism applied carfully. Humanlike shapes or features might provoke expectations that current technology cannot fulfil. Therefore, it was important to us that the robot is clearly identifiable as a machine; however, since anthropomorphic behaviors increase the usability of a robot, the SMOOTH-robot is equipped with some basic dialog and gaze behavior (Fischer, 2019) (see Figures 3, 4).

Given the complexities in human–robot interaction discussed above, it is unlikely that all problems will be solved within the next decade. However, using progress made so far in related disciplines such as vision, navigation, speech processing, and robot control, we believe that it is possible to realize basic interaction schemes, which might be sufficient for many tasks that are also relevant outside laboratories.

In the following, we describe some of the progress that has been made in different disciplines utilized on the SMOOTH-robot.

#### 2.2.1 Perception

Pose estimation and other vision processes have long been used in constrained industrial environments to perform manufacturing tasks, such as bin picking and packaging inspection. However, after the publication of AlexNet (Krizhevsky et al., 2012), deep learning has seen a surge in popularity, which has led to vision also being deployed in more unconstrained environments. While deep learning can be used to solve many types of problems, semantic segmentation and object detection are especially relevant for mobile robots. Semantic segmentation is the task of assigning each pixel in an image a label based on what type of object the pixel belongs to. This was first accomplished using deep learning in the study by (Long et al., 2014). Later models (Ronneberger et al., 2015; Chen et al., 2016; Chen et al., 2017) have improved both the efficiency and the accuracy on common semantic segmentation data sets. While semantic segmentation is very useful for differentiating between uncountable objects (stuff classes) like roads and walls, it does not distinguish between countable objects (thing classes) like humans and chairs. For this, object detection is used.

Object detection involves detecting each object of the desired classes and predicting a bounding box for each. Deep learning–based object detection algorithms are usually categorized into two categories: two-stage and one-stage detectors. Two-stage detectors (Girshick et al., 2013; Girshick, 2015; Ren et al., 2015) function by first using a region proposal step to propose regions of interest which could contain objects and then classifying those regions as objects/not objects and their class. One-stage detectors either use anchors, that is, they restrict detections to predefined bounding boxes and regions (Liu et al., 2015; Redmon et al., 2015; Lin et al., 2017), or are anchorless (Law and Deng, 2018; Duan et al., 2019; Liu et al., 2019) and omit the region proposal step of the two-stage detectors by going directly from input to classified bounding boxes. This greatly increases the speed of the detectors, albeit with a marginal reduction inaccuracy.

**FIGURE 5:**
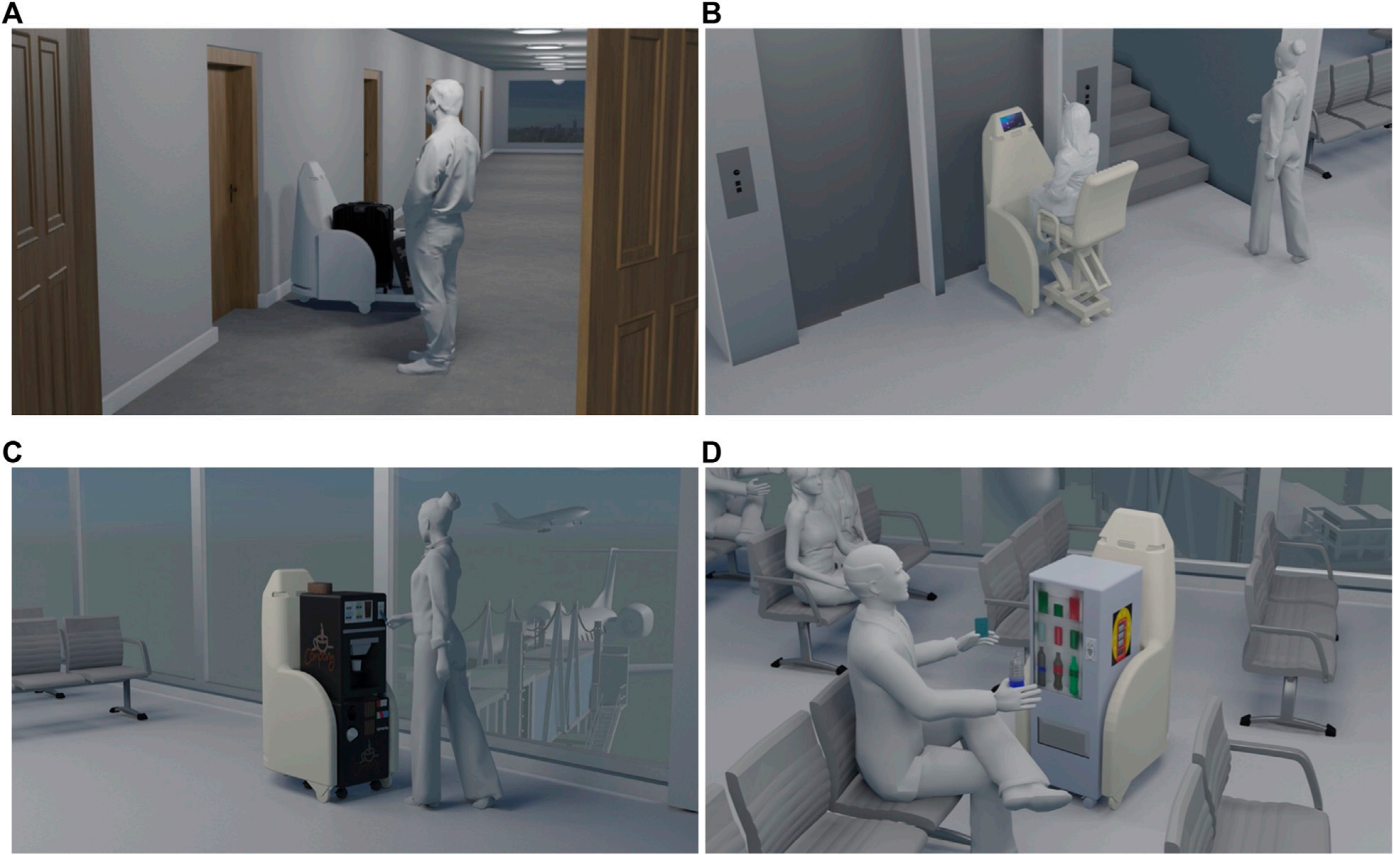
SMOOTH-robot solving four different tasks in various environments using four different modules: **(A)** baggage delivery in a hotel; **(B)** transport of humans in a hospital; **(C)** delivery of coffee; and **(D)** delivery of beverages in an airport.

**FIGURE 6:**
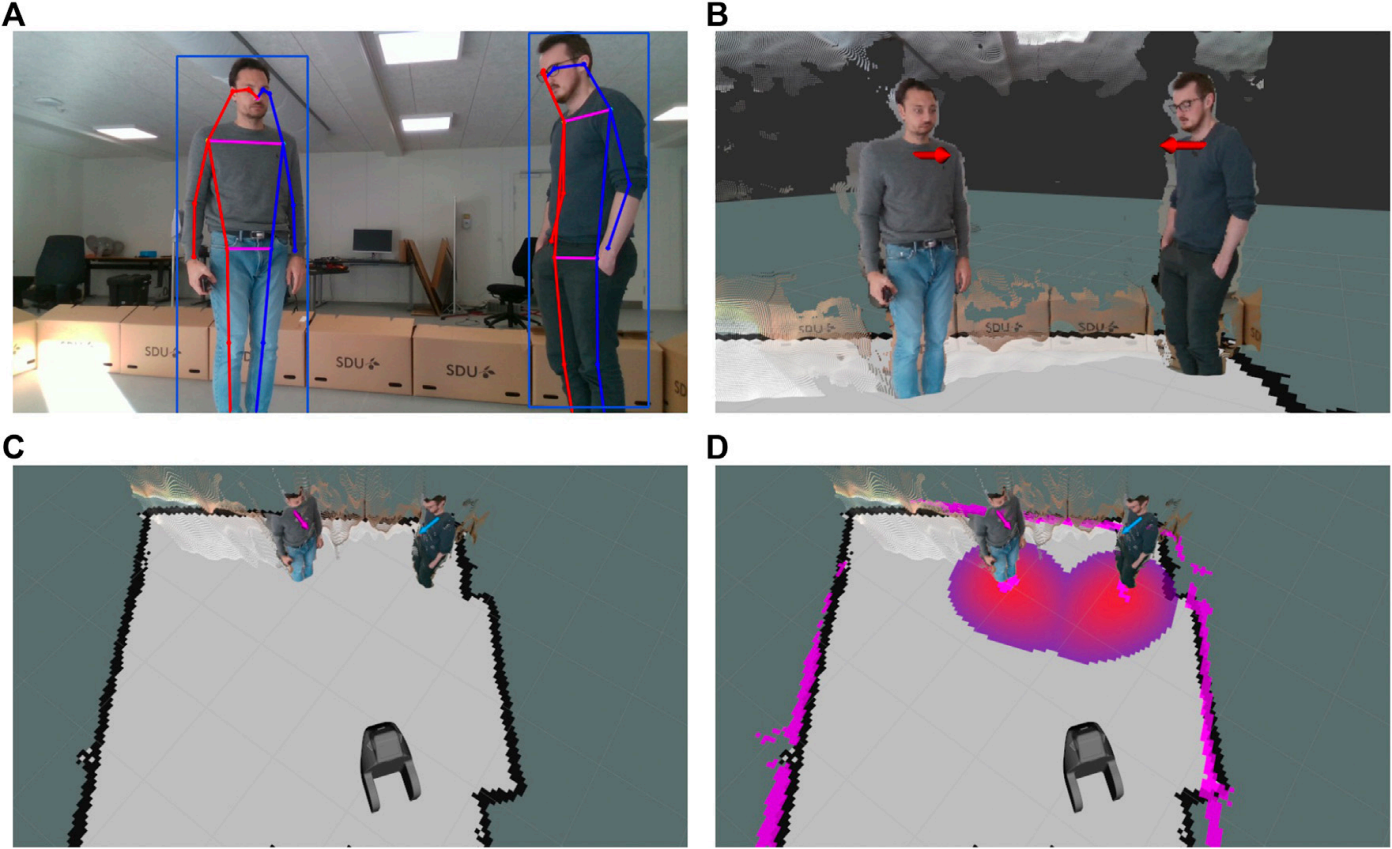
Visualization of the human pose detection module and the tracking-by-detection module with cost association: **(A)** output of the human pose detection network; **(B)** estimated torso position and facing direction; **(C)** assigned labels by the tracker (visualized with differing colors of the torso marker); **(D)** costmap with cost gradients assigned to each person.

These object detectors give a robot a good idea about which objects are in the environment, but they do not provide any fine-grained information since only the bounding box is estimated. To obtain a segmentation mask for each object, an instance segmentation network can be used. These exist in either accurate two-stage (He et al., 2017) or fast one-stage (Bolya et al., 2019) versions. Another type of object detector which estimates fine-grained information is the human pose estimator (Cao et al., 2018; Zhou et al., 2019), which estimates the skeletal structure of each person. In SMOOTH, we use the anchorless one-stage detector CenterNet (Zhou et al., 2019) to detect the add-on modules and people to solve the three use cases (see Figures 5, 6).

Gaze is an important nonverbal communication cue in human interaction. It carries valuable information on attention, intention, the emotional and cognitive state, etc. It can also be employed in human–robot communication for achieving a more natural interaction environment. A person’s gaze is not easy to estimate, especially from a distance, due to limited image resolution. This is why many authors in the past have resorted to using its first proxy, head pose (Doniec et al., 2006). However, actual eye gaze contains significant additional information, which can be utilized in human–robot interaction (Palinko et al., 2016). Recently, appearance-based gaze estimation methods have become more widespread, which allow easier remote eye tracking (Baltrušaitis et al., 2016). In the SMOOTH-project, we use this approach to make the robot’s behavior more responsive to people’s needs, for example, the SMOOTH-robot will offer a glass of water to the person in a group who establishes mutual gaze with it first.

#### 2.2.2 Navigation

Early indoor robot navigation followed two separate research tracks: metric (spatial) navigation (Cox, 1991; Figueroa and Mahajan, 1994) and behavior-based (topological) navigation (Arkin, 1990; Maja, 1992; Meng and Kak, 1993; Park and Kender, 1995). The spatial approach uses grid maps of the environment and relies on geometric pose estimates of the robot for planning and control, while the topological approach uses a graph representation of the environment and relational point of interest behaviors to transition from one node to another. However, the development of SLAM (Montemerlo et al., 2002; Durrant-Whyte and Bailey, 2006) and Monte Carlo localization (Dellaert et al., 1999; Thrun et al., 2001) resulted in the wide-scale adoption of spatial navigation in indoor robots. The use of spatial navigation was further boosted by the growing popularity of ROS middleware (Quigley et al., 2009), which provides open-source libraries for spatial navigation. Spatial navigation has proved to be very successful in static indoor environments; however, its strong dependence on the global geometric pose of the robot makes it unreliable in dynamic environments, where it is difficult to estimate the geometric pose of the robot. Furthermore, it does not incorporate semantic details of the environment, making it unsuitable for deployment in environments shared with humans. Current research in indoor robot navigation focuses on addressing these problems.

**FIGURE 7:**
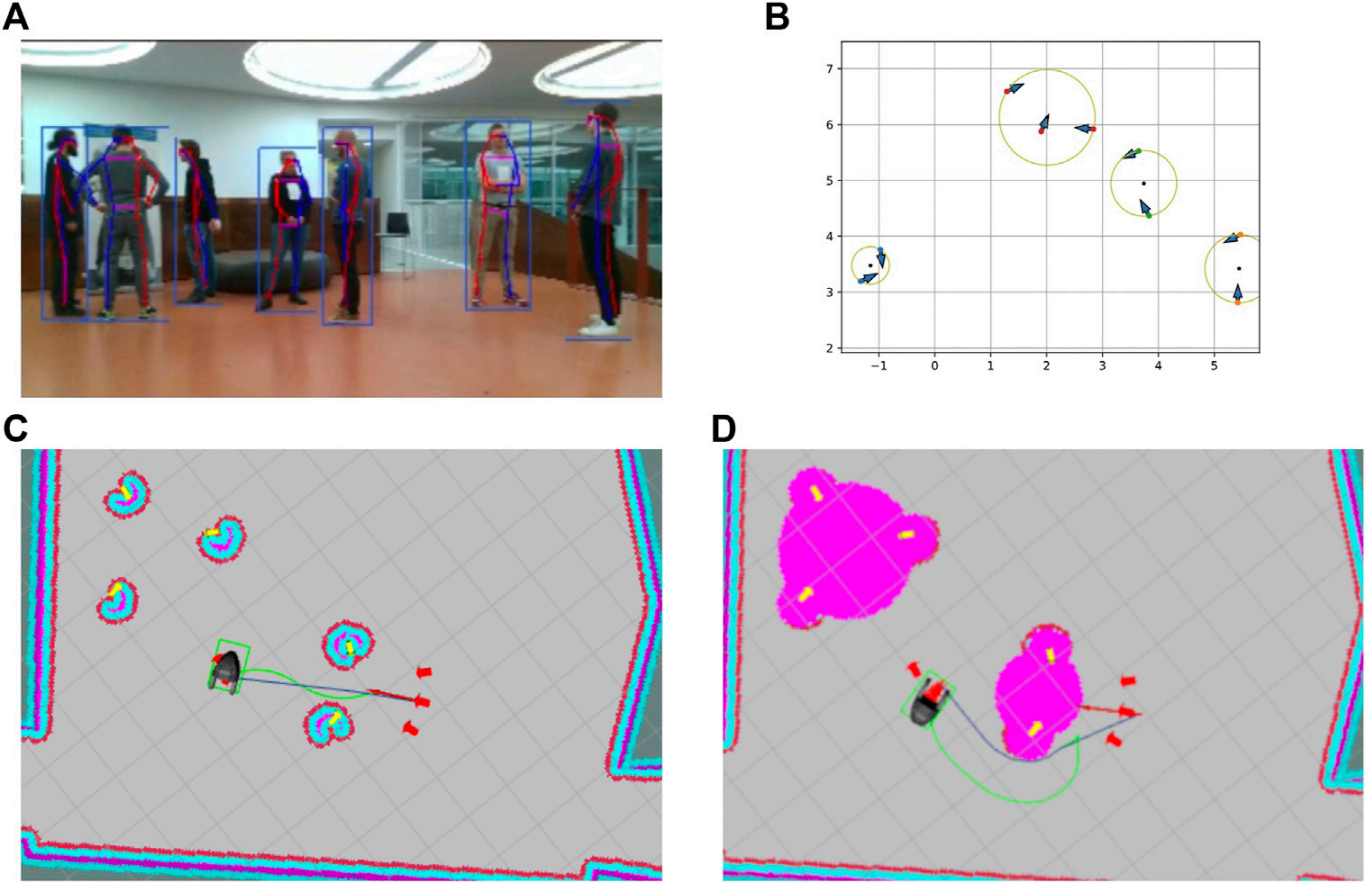
Grouping and socially aware navigation: **(A)** robot’s field of view; **(B)** detected groups, the blue arrows indicated the people’s poses, circles indicate the calculated o-space, and red arrows the optimal approach points; **(C)** robot motion planning using a standard obstacle-based costmap; **(D)** robot motion planning using an additional social costmap layer on the top of the obstacle-based costmap (yellow arrows indicate detected people, red arrows indicate planned approach points, the dark blue path indicates the global plan, and the green path indicates the local plan).

With the recently improved semantic perception capabilities (Zhao et al., 2019) of the robots, there have been several efforts to incorporate semantic details in spatial navigation with the use of semantic maps (Nüchter and Hertzberg, 2008; Kruse et al., 2013; Kostavelis and Gasteratos, 2015; Crespo et al., 2020). Semantic maps (see Figures 6, 7) enable robots to incorporate semantic details of the environment in their motion plans. As mobile robots in general are increasingly deployed in spaces occupied by humans, navigation techniques should also adapt to humans and accommodate their social conventions. Socially aware navigation studies the ways in which robots can move while respecting social norms while also allowing humans to feel comfortable. Respect of personal and social space, predictable navigation patterns, and reliable navigation patterns to increase comfort are investigated with multiple approaches, such as personal space modeling, the information process space (IPS) concept, and interaction spaces Kruse et al. (2013), Rios-Martinez et al. (2015), Charalampous et al. (2017a). Furthermore, increasingly, deep learning, and especially deep reinforcement learning are being used to learn socially aware motion plans (Alahi et al., 2016; Charalampous et al., 2017b; Truong and Ngo, 2017; Chen et al., 2019a).

Advances in deep learning have also resulted in the revival of behavior-based topological navigation approaches. Several works (Gao et al., 2017; Sepulveda et al., 2018; Chen et al., 2019b) have used end-to-end learning to learn behaviors for navigating in different types of indoor environments without a precise geometric pose estimate of the robot. While these approaches have shown promising results, they still lack the necessary libraries and tools for deployment on robots in real environments. Thus, in the SMOOTH-project, we have used spatial navigation and have extended layered costmaps [introduced in the study by Lu et al. (2014)] to model different social spaces retained by group structures such as F-formations to enable socially aware motion plans (cf. Section 4.3). This ensures that the SMOOTH-robot can be deployed in relatively static real-world environments along with social acceptance. Human–robot interaction should also be embedded in motion planning and execution in an appropriate fashion. In the case of the SMOOTH-robot, we integrated these two aspects within a behavior tree system (Colledanchise and Petter, 2018) and extended the robot capabilities to adapt to human motion using input correlation learning (Porr and Wörgötter, 2006).

#### 2.2.3 Speech

Robots are slowly learning to read human social signals and to coinhabit social spaces. Much progress has been made over the past decade with respect to speech (Moore, 2019) and social signal processing (Warta et al., 2018), safer navigation (Zacharaki et al., 2020), intention and activity recognition, etc. (Trick et al., 2019; Zhdanova et al., 2020). Regarding verbal interactions, considerable progress has been made in recent years due to the availability of hitherto unknown amounts of training data (Skantze et al., 2019). For instance, spoken interaction with computer systems such as Alexa or Siri is common nowadays; such dialog systems are trained on large databases of spoken interactions, storing common combinations of words and phrases and even question–answer sequences. The problem with such systems is that they operate on speech only and thus do not know what the words that they process mean. Thus, such systems are suited for chit-chat, but in order to get things done in the real world, dialog systems still need to be crafted by hand. Similarly, much progress has been made in speech recognition, but in spite of huge databases, speech recognition is still very bad at processing speech by novel users in noisy environments, especially if these users are younger [e.g., Kennedy et al. (2017)] or older [e.g., Zhou et al. (2016)] than the average user. Thus, speech recognition is one of the areas that constitute bottlenecks for the deployment of robots in social spaces.

However, there are many ways in which robots can support humans that do not presuppose sophisticated speech recognition; in particular, if robots can respond in a timely fashion to human behavior, then they can participate in social spaces. Thus, satisfactory interactions can be realized in robots as long as the robot is sufficiently responsive, which is what this project has focused on. Furthermore, people can also easily adapt to somewhat restricted interaction partners (such as children or non-native speakers,Fischer, 2016) by employing knowledge about interaction to the interpretation of the responses of limited interaction partners like robots; for instance, if a robot behavior occurs in response to a human request, people are likely to interpret this behavior as a reply. Thus, in this project, considerable work was carried out on coordinating speech and other robot behavior to make the robot responsive, timely, and interruptable [e.g., Baumann and Lindner (2015)]. We furthermore found the robot’s persuasiveness to depend on the coordination of speech and gaze behavior (Fischer et al., 2020a; Palinko et al., 2020).

The SMOOTH-project started off with a focus on the application of mobile robots in elderly care in the context of three use cases (see Figure 1). However, due to the Corona crisis, we could not experiment in the elderly care facility anymore and therefore extended our use cases, which turned out to be easier than expected since the developed robot competences could— due to the modular design of the robot—be easily transferred to other contexts, such as serving drinks at a reception or solving logistic problems at the university or other institutions.

## 3 The Design of the SMOOTH-Robot

With respect to the technological progress that has been made in the last decades and the still existing hurdles as outlined in Section 2, in the SMOOTH-project, we aimed at a reasonable balance between what is technologically possible and what is required to launch a new generation of service robots able to interact with humans. Our robot should neither require nor pretend to possess humanlike cognitive and social capabilities, which would only lead to unstable robot behaviors and user expectations that are doomed to be disappointed. Although we made a mild use of anthropomorphism, the overall design of the robot clearly indicates the presence of a machine and not a human or animal-like being and by that prevents such misconceptions.

The robot is also supposed to be producible to a price that can lead to acceptable business cases for end-users. Out of economic considerations, we decided that our robot should be able to solve multiple use cases outside the elderly care home also by applying different kinds of modules (see Figure 3).

### 3.1 The Design Process

**FIGURE 8:**
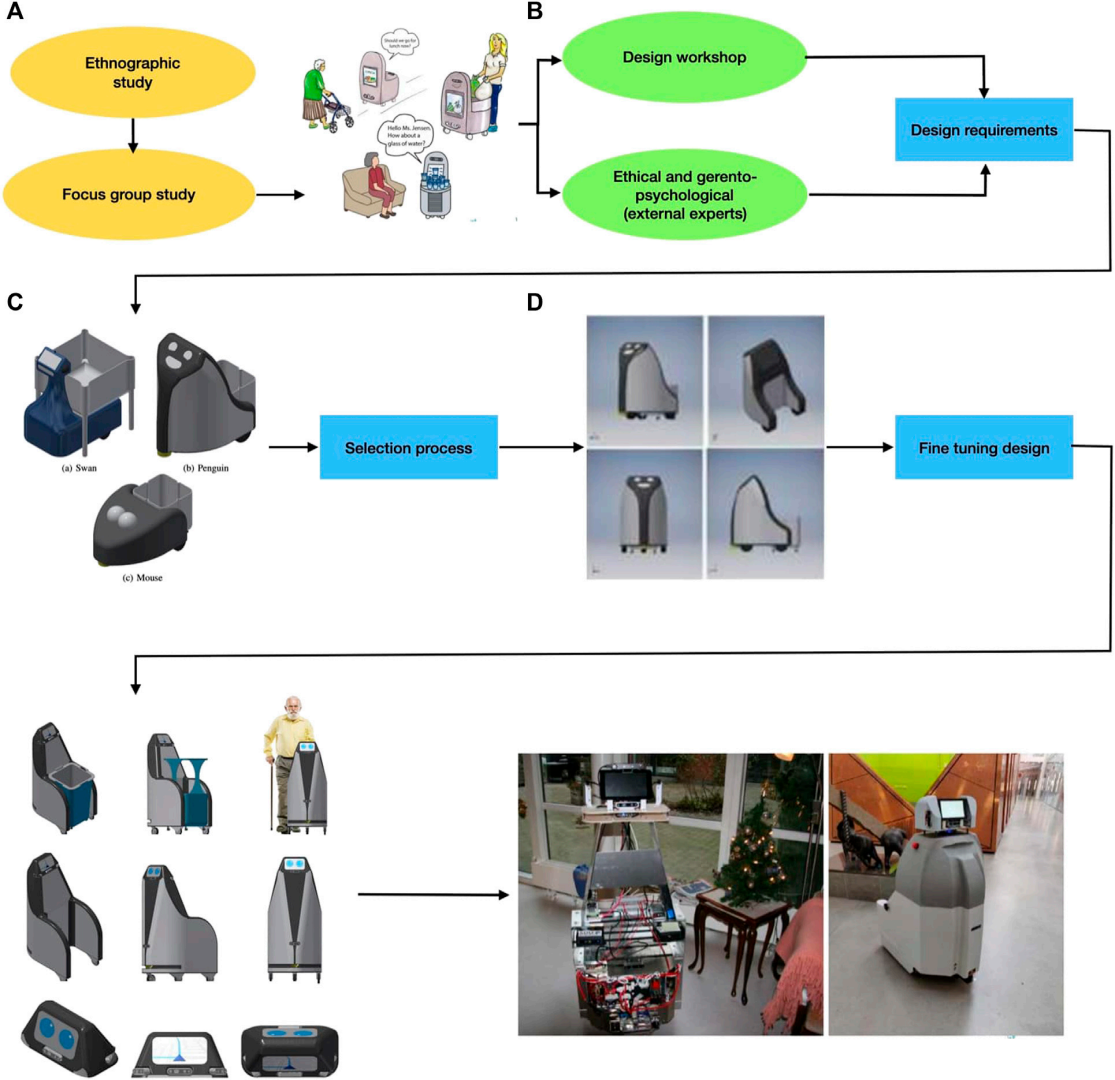
Overview of the SMOOTH design process: **(A)** ethnographic observation, **(B)** design workshop, **(C)** initial conceptualization, and **(D)** fine tuning.

The design process of the SMOOTH-robot [described in detail in the study by (Juel et al., 2019)] followed a user-centered design approach. We actively involved end-users, here care center staff and residents. The design process consists of four phases that are represented in Figures 8A–D. At the beginning (Figure 8A), we conducted ethnographic observation and situated interviews with caregivers and residents. From this study, areas where a robot could help the elderly and support the caregivers were identified, which then resulted in the definition of three use cases of the SMOOTH-robot, as also shown in Figure 1B.

To further include the end-user in the design process, we conducted a co-design workshop (Figure 8B) supported by situated interviews involving a focus group of caregivers and residents, during which the participants were asked to develop design concepts for a robot to solve the different use cases. To gain knowledge about how our robot would affect the end-user, we organized a workshop at a robot philosophy conference ^9^ where we invited external experts to discuss the use cases within the areas of ethics, design, and gerontopsychology. Some results of these discussions were published in the study by Fischer et al. (2020b).

Inspired by the user feedback, external experts, and the imposed requirements on price, shape, and functionality, the consortium designed three initial concepts for the SMOOTH-robot (Figure 8C). Each of the initial designs consists of the same three-wheeled mobile platform with two actuated wheels and a single caster wheel that allows the robot to turn around the axis between its driven wheels. In each design, the robot is able to pick up a module using different lifting mechanisms.

Within the consortium of the SMOOTH-project, we set up four main requirements for the robot design: affordability, modularity, simplicity, and acceptability. The three designs went through a selection process where the consortium discussed the shape, technical characteristics of the robot, and how well it would solve the use cases, with respect to the four main requirements. By means of this selection process, one design was selected for further conceptualization.

After having chosen the basic robot concept (Figure 8D), we made some refinements to the mobile platform, and we also designed a more specific UI hub (head). The refined version has the safety laser moved up above the wheels. To make sure that the full 270^*o*^ FoV of the safety laser is unobstructed in the placement, a groove was added to either side of it. The UI hub consists of screens, various computers, and vision sensors. Also, a first version of the different modules for solving the three use cases in the project was created.

Based on this refined design, a physical robot prototype was developed. The robot is internally built of three different parts. The bottom is a mobile base and includes the safety laser, batteries, motors, brakes, and other robot electronics and mechanics, which was built by the company Robotize ^10^. The batteries are placed along the sides of the robot, moving the center of mass further back. The middle part consists of all the processing units and a microphone used for speech recognition. The top part, the sensor head, hosts two screens and four stereo cameras used for human–robot interaction.

The design concept of the SMOOTH-robot is heavily focused on hardware modularity. Having a multipurpose robot where various modules can be designed and picked up to be transported by the same robot can create value in many different areas of society. Figures 3, 5 show some of the modules that were developed during the project to address different use cases. Furthermore, in Figure 2, different conceptual drawings are shown where the SMOOTH-robot uses different modules to solve different tasks in hotels, airports, and hospitals.

## 4 Sensorial and Bbehavioral Modules

The SMOOTH-robot is supposed to get involved in semi-complex interaction patterns making use of different sensorial modalities. This includes vision, in particular, the detection and interpretation of human actions (Section 4.1), basic navigation (Section 4.2) and socially aware navigation in environments where humans interact with each other (Section 4.3), the use of speech and dialogue (Section 4.4), and robot control (Section 4.5).

### 4.1 Visual Modules: Human Detection, Body Pose Estimation, Object, and Gaze Detection

In order for the robot to behave predictably in a human-rich environment, the robot needs to be able to detect where humans are in relation to it while estimating properties such as body pose, their walking speed and direction, and interactability. We developed a sophisticated vision system capable of such detections. The developed system consists of various vision modules to extract information from the environment and format the information so that it can be used by the robot to make decisions and perform actions. The core module is a multi-camera multi-detector tracking-by-detection system (Juel et al., 2020), designed specifically for mobile robots. It takes the output from any number of RGB-D sensors and processes it using a set of detectors, for example, the human detector shown in Figure 6A. The detections from each camera are then transformed into a common coordinate frame, allowing the tracker to operate on them collectively.

Any type of detector could be used in the system as long as it can output a 2D bounding box, a 3D position, and an optional 3D orientation. The detector developed in the SMOOTH-project uses a 2D convolutional neural network (Zhou et al., 2019) to detect objects in the RGB frame from each RGB-D sensor. The network in its standard configuration detects bounding boxes for many object types like a bed, chair, table, and human. This network is retrained to detect bounding boxes for the add-on module used for the logistic use case (see Section 5.1). It also has a configuration for human pose estimation where it simultaneously detects bounding boxes and joint positions for each person in the frame. Next, the detector projects each detection to 3D using the depth frame from the RGB-D sensor. If only a bounding box is available, the 3D position is found using the media depth value in a small square at the center of the bound box. In the human pose configuration, the position of the human is found as the mean position between the two shoulder joints. The heading direction is calculated as the vector orthogonal to the vector between the shoulder joints (Figure 6B), since people tend to position their torso in the direction they are moving.

The next step in the tracking-by-detection system is assigning a temporally consistent ID to each detection using the tracker (Figure 6C). With this, it is possible for the robot to differentiate between people while estimating their velocities and thereby predicting their future position. Tracking people’s position enables the robot to maintain a temporally consistent costmap (Figure 6C). The tracker makes the costmap consistent even in the event of occasional false negatives where a person is not detected for a few frames or if they leave the field of view of the camera, by predicting where they are going. Another feature of the tracker is that it enables the robot to perceive the detected features of a person over time. These features include the skeletal pose of the person, which, as a time series, could be used for action recognition. The detectors and tracking-by-detection modules are used to detect people to interact within the guiding and drink-serving use cases (see Section 5.2 and Section 5.3).

Another important feature that the robot can use to make decisions is the person’s gaze. There are two aspects of gaze interaction that are important for the SMOOTH-robot: analysis of humans gaze and display of the robot’s own gaze to communicate intention.

**FIGURE 9:**
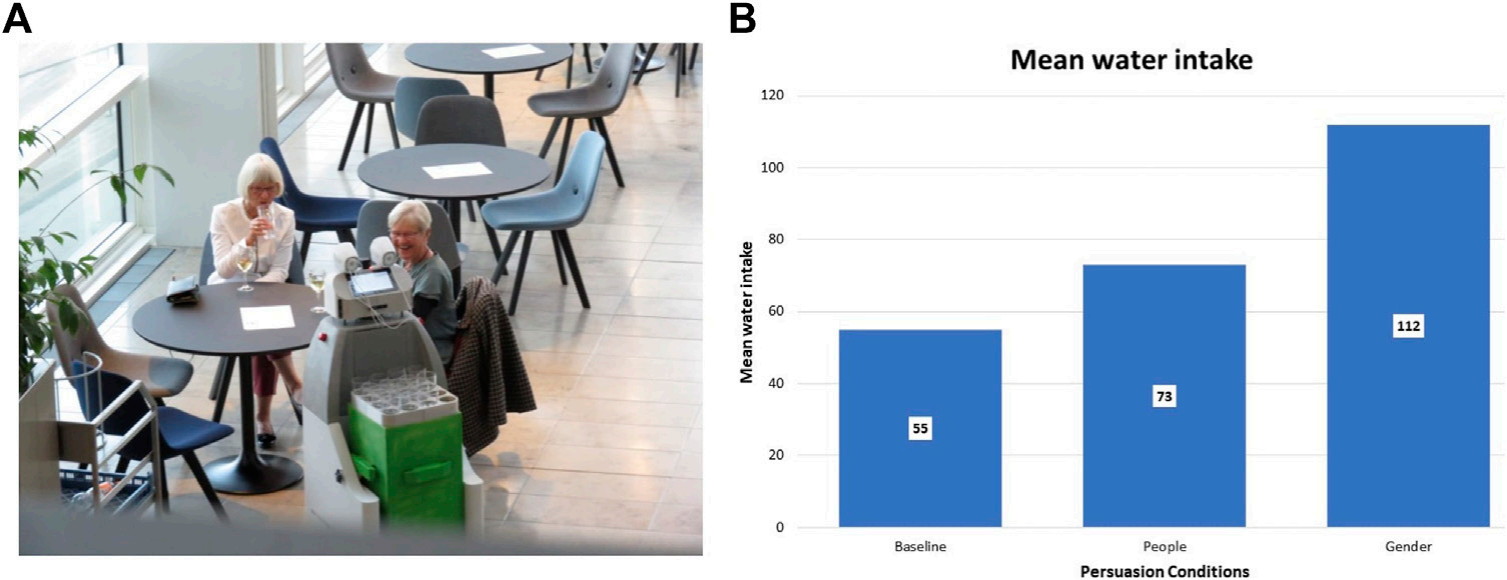
Persuasive robot dialogue; **(A)** robot’s persuasive utterances successfully encourage members of the general public to drink more; **(B)** persuasive dialogue (“most participants” or “most female/male participants”) leads to significantly higher water intake.

We use the open-source appearance-based head and gaze-tracking software library, OpenFace (Baltrušaitis et al., 2016) (see Figure 9A), for analyzing people’s gaze. It estimates people’s head pose and gaze, which are quite usable in interaction scenarios. Knowing where people look can enable the robot to be more reactive to their attention. Mutual gaze is a special interaction cue that signifies that two interaction partners are looking at each other and are aware of this shared visual attention. If there is mutual gaze between a person and the SMOOTH-robot, it is safe to assume that the person is attending to the robot, and thus, the robot can start talking to him or her (see Figure 9B). Conversely, if the human is looking elsewhere, the robot knows that it is not yet time to start a dialogue. In this case, the SMOOTH-robot can try to capture the person’s attention by either looking at them silently or by speaking while gazing at them (“Excuse me, do you have a minute?”). Technically, the gaze tracking algorithms estimate the head pose and the gaze angle of humans, which provides gaze vectors in 3D space. If these vectors intersect with the robot’s body, it is considered that people are looking at it. As SMOOTH proceeds with the interaction once this signal is detected, gaze interaction makes the robot more responsive and more interactive.

Regarding the robot displaying its own gaze, we are simulating two eyes on the robot’s front display (see Figure 9C). The eyes are designed in a very symbolical and abstract way; they are shown as two large white circles on a gray background representing the scleras, which contain two small black circles, representing the pupils. The pupils move on a white background to simulate the robot’s gaze direction. It also blinks from time to time to make the gaze behavior more natural (see Figure 9D). When the robot’s cameras detect faces in front of the robot, it will start looking at them. It will switch between the different detected faces, thus simulating human gaze behavior. It also breaks eye contact with people once in a while, as it is unnatural to keep staring at a person.

One important task of displaying gaze on the SMOOTH-robot is serving the purpose of conveying intention; SMOOTH communicates to its environment what its visual system is focused at. This has been proven very useful in selecting whom the robot will interact with next (Palinko et al., 2020). It has also been found that the robot can be more persuasive when establishing eye contact with its interaction partners (Fischer et al., 2020a).

### 4.2 Basic Navigation

The SMOOTH-robot utilizes and builds on the top of the navigation stack in ROS (Marder-Eppstein et al., 2010), often referred to as *move_base* or the navigation stack. A prerequisite for using this framework is that the robot has recorded a map of the environment, using any type of SLAM (simultaneous localization and mapping) algorithm and can localize itself inside this map during the operation. The robot accomplishes this by utilizing its sensor suite comprising odometry encoders, an IMU, and a laser scanner.

The static map, in which the robot can now localize itself, is used as the basis for the search space of the navigation algorithms that calculate the robot’s route. In *move_base*, this search space is made up of a layered structure of costmaps, that is, maps that describe the cost of being at a certain location due to some specific information about the environment, for example, static objects, dynamic obstacles, people, social constructs, and more.

**FIGURE 10:**
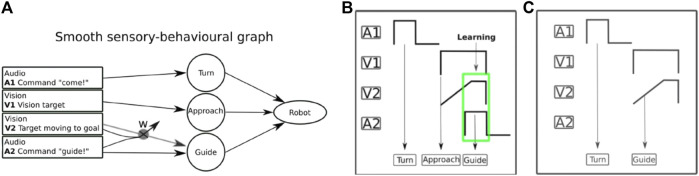
Learning transitions between behaviors in use case 2: **(A)** sensory-behavioral graph in the transition of guiding when the user approaches the robot. A1, V1, V2, and A2 are sensory inputs of the SMOOTH-robot. A1 is an audio input (i.e., human command “come here!”). V1 is a visual input (i.e., human face recognition). V2 is another visual input (i.e., human motion detection). A2 is another audio input (e.g., the human command “guide me!”). *W* refers to an adaptive connection that will be established after learning. It will transmit a predictive signal (i.e., human approaching detection) to activate the guiding behavior. **(B)** In the first iteration, the robot perceived the first audio input and turned toward the user call or sound source (A1 → Turn). After that, the robot visually detected the user and then approached (V1 → Approach). Then the robot detected the human motion (i.e., V2, human walking toward the robot as a predictive signal) and thereafter human speech (i.e., A2, guide me! as a reflex signal) which finally leads to robot guiding behavior (A2 → Guide). In this process, the robot also learned that the inputs V2 (predictive signal) and A2 (reflex signal) are correlated (learning process), meaning that the human motion implied a need for the guiding behavior. **(C)** After learning, the robot first perceived the sound and turned toward the sound source (A1 → Turn). Afterward, it detected the human (V1) and his motion (V2). It then immediately performed the guiding behavior without its approaching behavior. In this process, the robot basically proactively skipped its approaching behavior. In other words, the robot will perform guiding behavior as soon as it detects the movement of the human toward a given target location (e.g., the dining room).

The path planning algorithm searches for a path from point A to point B in the combination of these costmaps by optimizing for the lowest cost. Figure 10 shows the robot positioned in a tight corridor and facing upward but being asked to go to the position of the red arrow behind it. In Figure 10A, the global path planner is using Dijkstra’s algorithm to plan the shortest path, but this results in the robot being asked to turn on the spot in the tight corridor, which is not possible. Figure 10B illustrates another path planning algorithm called SBPL or Search Based Lattice Planner (Likachev and Ferguson, 2009), which utilizes knowledge of the precise footprint and kinematics of the robot to plan a path that is feasible for the robot to follow. Planning using SBPL is more time consuming but results in paths that are kinematically and dynamically feasible; for instance, it would not plan a path that tells the robot to rotate on the spot if there is not enough room for the robot to physically do so. Therefore, it depends on the use case and the environment of the robot as to which algorithm is the most suitable. The SMOOTH-robot is configured using both planners and can use either depending on the actual situation; for example, fast planning times are preferable in a dynamic environment with many people.

There are two types of costmaps in the navigation stack—global and local. The global costmap covers the entire area of the robot’s environment and is utilized by the path planning algorithm. In contrast, the local costmap is constrained to a local area around the robot and follows it around. This reduced the computational effort in keeping the local costmap up to date with the newest sensor information, which is coming in at high frequencies from the laser scanner and the camera. The local costmap is utilized by the controller of the robot that calculates the velocities for the robot wheels that allow it to follow the planned path. In Figure 10, the global costmap is shown in grayscale and covers the entire area—notice the gray gradient around the black walls. The local costmap is shown in color and is centered on the robot and shows a similar cost gradient around the walls.

Due to the layered structure of the costmaps, it is possible to influence the planner behavior by simply creating a new layer that describes a certain type of cost, such as the cost of social spaces and grouping described in Section 4.3.

### 4.3 Socially Aware Navigation

Humans instinctively use social cues, such as facing direction, occupied space, and body language, to deduce whether others are interacting, thus avoiding interruptions and allowing them to join the activity. Such interactions are common and consistently exhibit certain arrangements. F-formations (Kendon, 1990) describe the distinct group structures that spontaneously form when two or more people are interacting and the social areas constructed by the groups. These occupied areas are defined as follows: the o-space, the area within the group, reserved for interaction, the p-space, the area incorporating the previously mentioned space and the persons’ bodies, and, finally, the r-space, which is the surrounding space and is reserved for individuals joining or leaving the group. The o-space can be seen in both Figures 7B,D.

On our robot, based on the vision modules described in Section 4.1, an agglomerative hierarchical clustering method is used to detect social groups using the persons’ positions and orientations, thus considering the social structures mentioned before (Kollakidou et al., 2021). The individuals are clustered with a criterion of a maximum distance allowed in case of intended interaction. The persons’ orientations are used to positively influence the distance function used for clustering for individuals who are facing each other or share the same focus point and negatively influence the distance function in cases where they do not share a field of view. Figure 7A shows individuals whose poses are detected interacting, and Figure 7B shows the detected groups and calculated approach points. The robot can then approach the groups without disrupting the interaction or altering the group’s structure. This is achieved by determining the o- and p-spaces and avoiding crossing the former while attempting to enter the latter and thus acting as a group member.

As standard navigation techniques (as described in Section 4.2) do not consider all of the previously mentioned cues and restrictions, they may result in motion patterns that are perceived as uncomfortable and suboptimal by humans, indicating the need for socially aware navigation. Socially aware navigation aims at incorporating all static and dynamic parameters of the environment and making informed decisions considering the outcome of the robot’s actions. To enable socially aware navigation, we introduce an additional costmap layer on the top of the standard obstacle-based costmap to model the p-space and the o-space of the detected people and detected F-formations.

Figures 7C,D describe the difference between the motion plans generated by basic navigation and socially aware navigation. In the given example, the robot is navigating from the first group (a group of 3 people) to the second group (a group of 2 people). The robot starts with the people grouping according to the algorithm described above and adds the o-space of the detected groups and detected people to its costmap as shown in Figure 7D (pink-colored cost around the detected people). Figure 7C describes the robot’s motion plan for moving from the first group to the second group using the basic navigation (see Section 4.2). Here, the robot uses the costmap based on obstacles and an inflation radius of 25 cm around the obstacles for motion planning. The global plan is generated using a Dijkstra planner, while the elastic band local planner (Rösmann et al., 2017) is used for planning the online motion based on the dynamics of the robot and the global plan. As shown in Figure 7C, the robot ends up planning the shortest path without respecting the social and personal space of the surrounding people.

Figure 7D describes the robot’s motion plan with socially aware navigation. Here, the robot uses the costmap which not only incorporates the detected obstacles but also the social and personal space of the detected people, thus resulting in the socially aware motion plan.

### 4.4 Spoken Interaction and Dialogue System

Once a human has been successfully approached as described in Section 4.3, a dialogue can unfold as an exchange of speech utterances in turns. Many dialogue systems model this as a back-and-forth exchange. However, turns may overlap, listeners may interrupt a speaker, and the speaker may consequently adapt or abort their utterance while it is being spoken, based on her perception of the listener or the environment. For example, the environment may change and additional knowledge may be gained by the SMOOTH-robot that may lead it to change its speech plan while it is speaking. Such behaviors are summarized as incremental dialogue processing (Baumann, 2013), and we have previously shown that incremental adaptation of speech behaviors based on how the situation evolves improves the perceived sociability of a robot (Baumann and Lindner, 2015).

Given the problems with recognizing speech of users, and especially older adults [see Zhou et al. (2016)] in noisy environments, we focused on implementing robot responsiveness into its speech output behaviors. Incremental speech output production requires incremental speech synthesis (Baumann and Schlangen, 2012b) so that speech output can be seamlessly extended (or shortened) without audible breaks. In the project, we extended the existing state-based dialogue system DialogOS^11^ (Koller et al., 2018) in two ways: we integrate incremental speech input and output capabilities based on InproTK (Baumann and Schlangen, 2012c) and we integrate an ROS interface to enable the tight coupling between robot behavior and robot speech and dialogue behaviors. This system allows for dialogue models that feature interruptability and adaptability based on unexpected events (such as an obstacle or potential danger), but also any other sensory information, such as when the person being guided has disappeared from the robot’s camera view. Furthermore, the system allows for the incremental synthesis of robot utterances, thus preserving the prosodic integrity of utterances (Baumann and Schlangen, 2012a), even when they are interrupted, and the smooth adaptation of the robot’s loudness of speech (Rottschäfer et al., 2015) depending on the distance of the addressee. Besides enabling dialogue, this also allows for incremental monologue, that is, speech synthesis that is adapted to the external context. Monologues and dialogues were implemented for the guiding use case (see Section 5.2) with positive content, that is, some emotionally nonarousing comments of potential interest to the target community (such as what is planned for lunch, whether there are new animals at the local zoo, etc.) based on our use case development results in the study by (Fischer et al., 2020b).

Furthermore, we developed SMOOTH-robot utterances that rest on shaping participants’ replies and thus predicting their next utterances to circumvent speech recognition bottlenecks. We created persuasive robot dialogues in the context of the beverage service use case (see Section 5.3) that have been demonstrated to lead to significant behavioral effects; in particular, since dehydration is a considerable problem in elderly care facilities, we concentrated on increasing people’s water intake, and our studies show that the persuasive dialogues we created lead to significantly higher water intake than the baseline dialogues.

For instance, one of the persuasive strategies we experimented with is the personalization of social proof, where the robot appeals to other groups that serve as an example for people’s choices in the current situation. Corresponding to findings by Goldstein et al. (2008), we find that tailoring social proof to the gender identity of the participants leads to more than twice as much water intake as in the no-persuasion condition, in which people were only informed about the benefits of water intake, and the general social proof condition.

**FIGURE 11:**
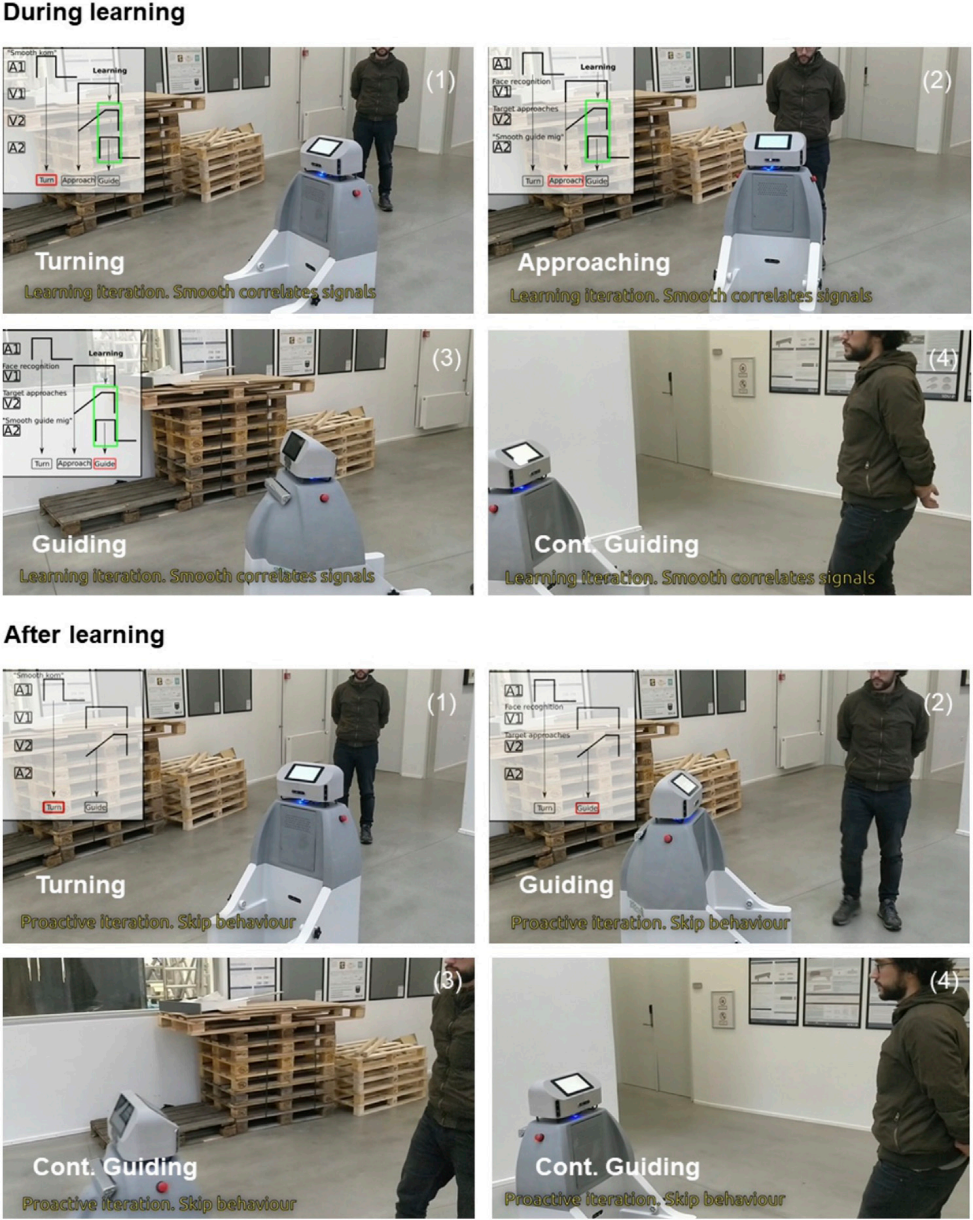
Snapshots of robot learning transitions. In the learning phase, the SMOOTH-robot performed three sensor-driven behaviors sequentially: turning driven by A1 (audio feedback), approaching driven by V1 (visual feedback), and guiding driven by A2 (audio feedback). Simultaneously, in this phase, the SMOOTH-robot also learned the correlation of a visual-based (earlier) predictive signal (V1) when seeing the human walking to it and an audio-based (later) reflex signal (A2) when hearing the command asking it to guide. After learning, it can realize the human motion (walking toward it) as the human intention to walk to a destination without naming it explicitly. As a consequence, it switched to its guiding behavior to guide the human.

In this experiment, which was carried out both in our laboratory and in the community’s LivingLab, the robot guided participants through the laboratory and instructed them to pick up things to set a table. In the course of the experiment, the robot mentions the benefits of water intake and then either uses a specific persuasive utterance or not, which allows us to measure the impact of a single persuasive utterance on water intake. That is, at the end of the experiment, participants sit down at the table they have set themselves, which includes a glass and a jug filled with water, while they fill out the postexperimental questionnaire. After the experiment, we measured how much water was missing from the jug and their glass. Our results, illustrated in Figure 11, show that people drank significantly less water if there was no further persuasive utterance than they did if the robot mentioned that “most female/male participants drink half a liter after this game” (depending on the respective participant’s gender), with the general message “most participants drink half a liter after this game” being in the middle. These and similar persuasive utterances were also successfully tested in the wild (see Figure 11B) where the robot offered water to members of the general public (see Figure 11A), including many older adults (Fischer et al., 2020a; Palinko et al., 2020).

The dialogue system developed has also been used in the guiding use case (see Section 5.2), using incremental processing to adapt to the time it takes to reach the destination, to respond to interruptions in a timely fashion, and to adjust the robot’s loudness to the distance from the respective user. Incremental robot response can also be used to help people find objects by taking their current actions into account, for instance, in “left, a bit further, yes, there!” Baumann et al. (2013). Such an utterance depends on the user’s current behavior Jensen et al. (2020). Because the dialogue system developed specifies not only the dialogue flow but also the interaction with robot behaviors through the integration with ROS, speech can be adapted on the fly (i.e., continuously despite the state-based model) to sensory information from the robot. In the guiding use case, this feature is used to, for instance, greet other residents (in the elderly care facility) or other people in the corridor and then to return to the small talk during the joint walk.

### 4.5 Adaptive State Transition Model

The modules described in Section 4.1, Section 4.2, Section 4.3, and Section 4.4 are integrated into behaviors that are then sequenced and executed to generate the tasks required in the use cases that are presented in Section 5. The organization and sequencing of the behaviors is done through behavior trees (Colledanchise and Ögren, 2017), which generalize hierarchical finite state machines in a modular way. Although originally created to generate reusable behaviors of nonplaying characters in games, behavior trees have also been applied to create complex tasks in robotics, and they have been shown to encompass other control architectures like those using state machines. State transitions are implemented as the predefined execution order of the nodes of the behavior trees, which provide a fixed execution pattern for the implemented behaviors. In our case, however, we included an adaptive execution model based on learning, which can skip behaviors on the tree (equivalently, states of the corresponding state machine) to make the robot behavior more fluid. Specifically, we used the adaptive execution model in use case 2 (see Section 5.2), where we integrated two sensory modalities of the robot (vision and audio) through a correlation-based learning mechanism [see Shaikh et al. (2019) for more details of the mechanism] to create state transitions between robot behaviors with pro-activity. In the context of use case 2, guiding to the dining room (goal), the SMOOTH-robot performs several behaviors with transitions in the following sequence:

Step 1: turning toward the direction of the call (i.e., the user (caregiver) calls “SMOOTH come here” and the SMOOTH-robot hears the call),
Step 2: approaching the user (i.e., during turning, the SMOOTH-robot uses the vision modules described in Section 4.1 to detect the user’s face and starts to move toward the user, while at the same time, the user may also walk toward the SMOOTH-robot to prepare (user approaching) for following the SMOOTH-robot to a destination),
Step 3: guiding the user to the destination (i.e., the user tells the SMOOTH-robot to guide him or her to the destination).

**FIGURE 12:**
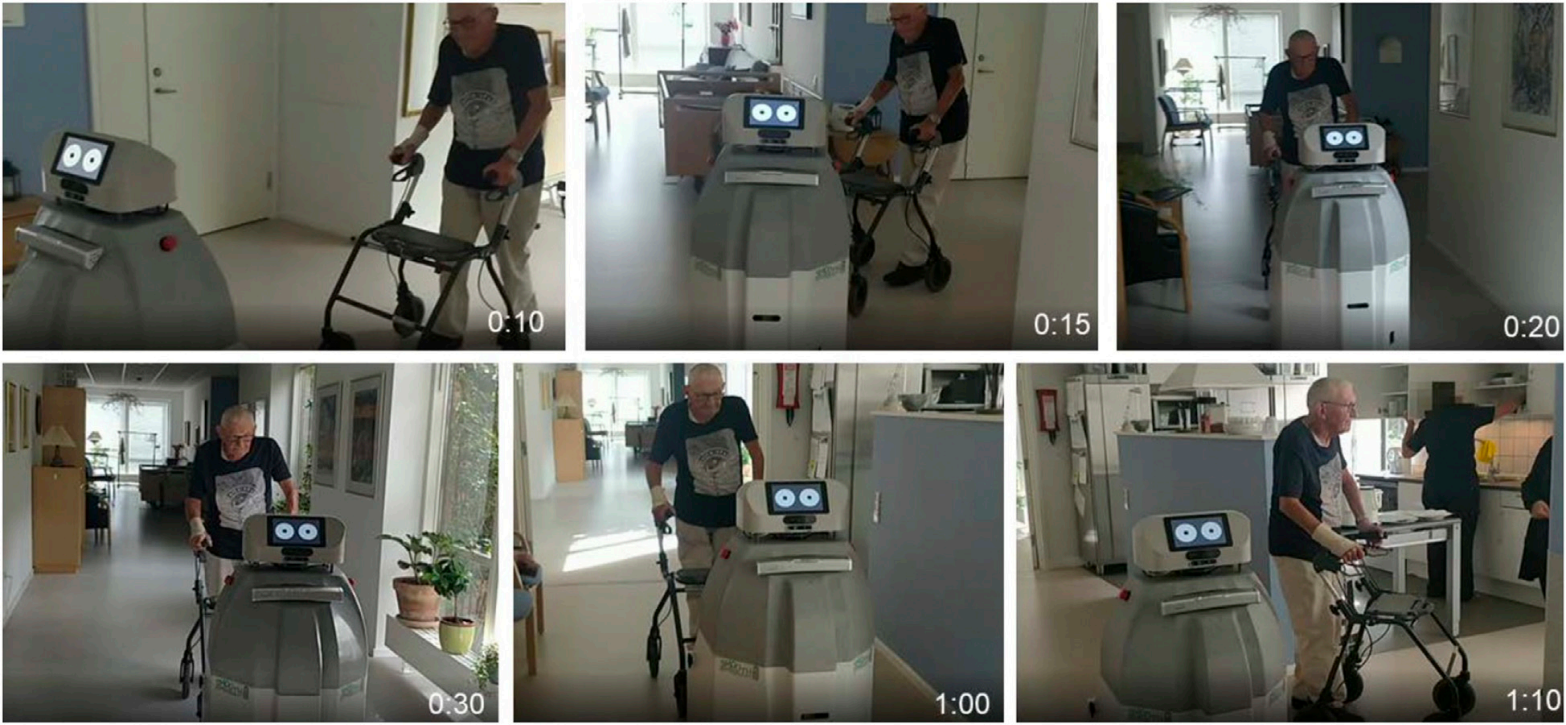
SMOOTH-robot successfully guided an elderly resident at the Ølby elderly care center in Køge, Denmark. It autonomously navigated in the center and smoothly guided the resident without a stop-and-go pattern from the living room to the dining room over a distance of 25 m. During guiding, it also adapted its speed to the human target for effective guiding. Furthermore, it also performed incremental monologue to encourage the resident to walk to the dining room.

For learning state transitions in this scenario, the correlation-based learning mechanism will learn a new proactive behavior transition in the human–robot interaction by correlating predictive (earlier) and reflex (later) signals in the sensory-behavioral graph (Figure 12A). The proactive behavior transition is learnt when the reflex and predictive signals overlap. After learning, the new pathway of the predictive signal will be created to drive the proactive behavior. On the SMOOTH-robot, this mechanism is implemented as follows: we use computer vision (see Section 4.1) to detect the user approaching, which provides the predictive signal and the audio command recognition as the reflex signal of the robot guiding behavior. In the first iteration, the transition of the sensory-behavioral connection does not exist. When both signals are overlapping as shown in Figure 12B, the connection is learnt. In Figure 12B, a new interaction with the robot occurs with the new transition. The recognition of the user approaching drives the activation of the guiding behavior before robot approaching behavior occurs. The control takes over this behavior, and there is no need for command recognition. With this adaptive state transition model, we show the flexibility of the system where the SMOOTH-robot can interact in a normal way in the following behavioral sequences: 1) approaching and 2) interacting toward guiding verbally with the user. Under the learning state transitions, the SMOOTH-robot can also skip approaching and directly perform guiding behavior if the SMOOTH-robot anticipates human movement toward the dining room through the vision-based (earlier) predictive signal. The learning of transitions has been successfully tested in use case 2 (guidance scenario).

**FIGURE 13:**
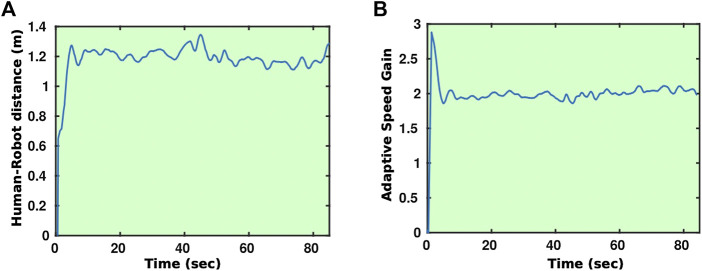
Time evolution of the human–robot distance **(A)** and adaptive gain **(B)** during a guiding experiment.

Figure 13 shows the result of the real human–robot interaction experiment. During learning, the robot performed 1) turning, 2) approaching, and 3) guiding. After learning, the robot can use predictive visual feedback (V2, Figure 12) to detect human movement early. In this experiment, in the second repetition, the human moved toward the destination without waiting for the robot to approach. The robot detected the human movement; thereby it switched to guiding instead of approaching as can be seen in a video.^12^

## 5 The SMOOTH-Robot Applied to Three Use Cases in Elderly Care and Beyond

In the following three subsections, we will describe the application of the SMOOTH-robot in the context of the three use cases discussed before (see Figure 1B).

### 5.1 Logistics

The logistic use case of collecting laundry and garbage bins makes use of the sensorial modules described in Section 4.1, the navigation behavior described in Section 4.2, and an additional object detection neural network for detecting bins. The use case flow is split into 6 steps:

1. The robot navigates around the area searching for bins that have to be transported. This is currently indicated by the bin’s position (e.g., left of the entrance of a door to a room of a resident), but it could also be indicated by some kind of active IFID signal.
2. The robot detects a bin *via* the trained neural net (see Section 4.1) and estimates an initial bin position in the map. We use some of the methods explained in the study by Haarslev et al. (2020) to create a bin pose estimator using an object detection network (Zhou et al., 2019).
3. The robot navigates toward the initial position, while still detecting the bin until a stable pose is measured using the very same neural network.
4. The robot moves according to the measured stable pose and aligns the backside with the bin. The robot chamfers at the end of the two robot back wings and can compensate for some uncertainty in the pose estimation process.
5. The robot docks the bin and lifts it up using an automated lifting mechanism (Figures 5C,D). After that, the robot can freely drive away with the bin and automatically drop it off again at a designated drop-off spot.

The first iteration of the use case follows the above procedure and was executed at an elderly care facility. In this iteration, we still used markers for detection and pose estimation of a wooden prototype of the bin which had to be manually put on the robot by a human since there was no automated lifting mechanism. The final iteration of the use case was executed in the hallway at the University of Southern Denmark. A video of both iterations is available ^13^.

### 5.2 Guiding

**FIGURE 14:**
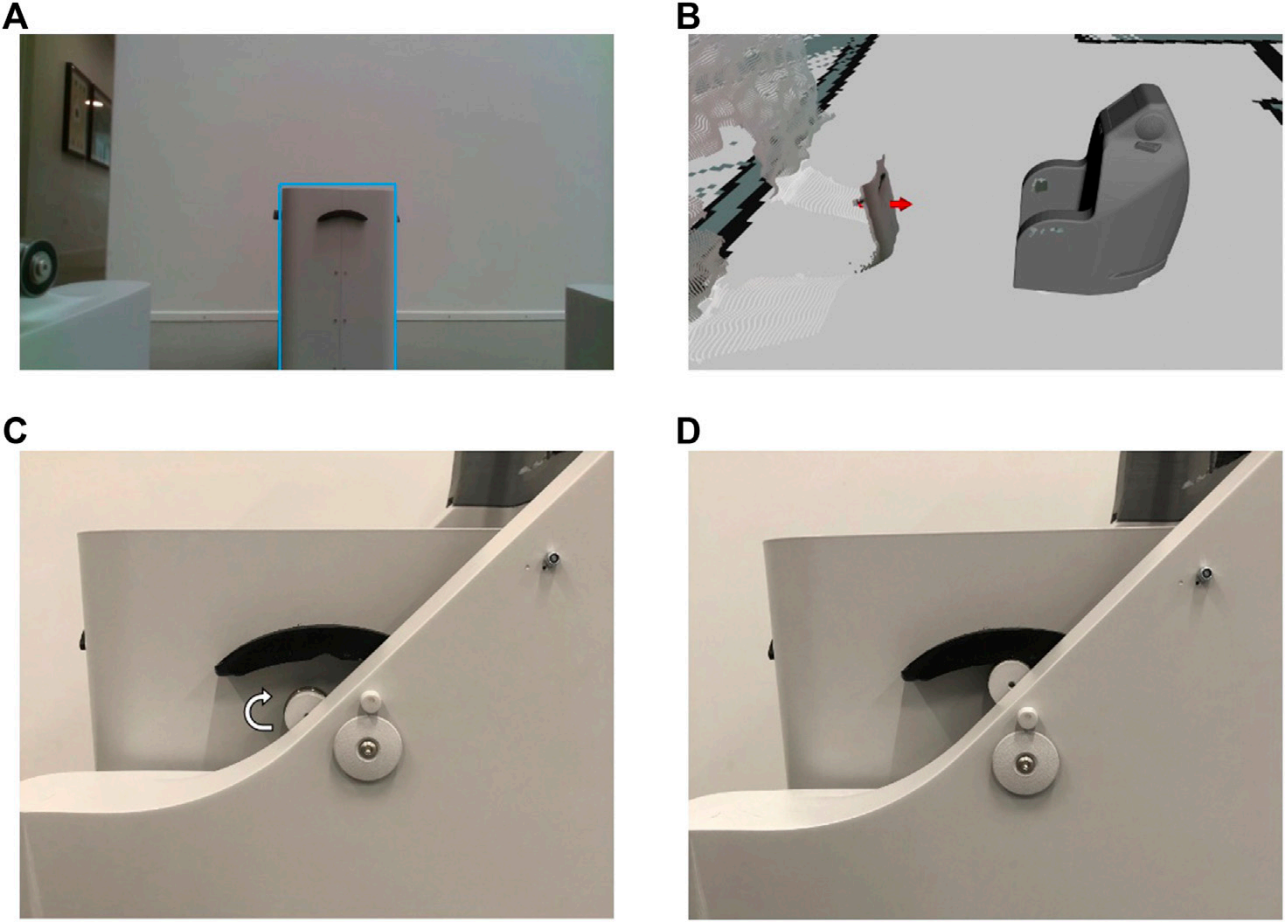
**(A)** Bin detection (blue rectangle) and **(B)** pose estimation (red arrow) during the docking behavior. **(C,D)** Lifting mechanism on SMOOTH.

Due to demographic change, health and elderly care systems are dealing with a shortage of qualified caregivers. To address this, we introduced a SMOOTH-robot to an elderly care center. Based on our needs analysis, one of the important tasks for SMOOTH is to guide an elderly resident to navigate to a target location (e.g., from the living room to the dining room) in the center. The SMOOTH guiding function includes several behavioral sequences: turning, approaching, and guiding/navigating [see details in Section 4.5 (steps 1–3)]. To achieve this complex task, we developed adaptive modular-based guiding control software and implemented it on the SMOOTH-robot (Figure 14). The software consists of the following sub-modules: incremental monologue, turning, approaching, guiding, and navigation. It can generate robot proactive behaviors with human–robot dialogue and incremental monologue to smoothly interact with and guide elderly people to the dining room in the elderly care center.

A key component with respect to the guiding of the residents is the adaptation of the speed of the robot to the speed of the person since the residents may have quite different movement capabilities. The navigation process was extended using a mechanism that adapts the gain of the robot speed during the planned trajectory following to the walking speed of the resident. Figure 4 shows some examples of a guiding experiment in which the robot adapts its speed to match the speed of the human it guides. As can be seen from Figure 4A, the robot stays within ±25*%* of a predefined distance. Figure 4B shows the value of the speed controller gain as a function of time throughout the guiding process; as stated before, the gain is adapted to ensure the robot matches the speed of the human.

The incremental monologue, that is, the incremental synthesis of robot utterances depending on sensory data (see Section 4.4), is implemented in such a way that the robot adjusts the small talk produced in this situation, for instance, about the lunch menu or other topics of possible interest to the person guided, to the time it takes to arrive at the dinner table. We can also tell jokes to the resident where various joke lengths are estimated and selected online based on the remaining distance to the destination (i.e., the dining room). For example, the robot can tell jokes where a short joke will be selected if the remaining distance is short. The result of this implementation is demonstrated as use case 2. We tested the control at the Ølby elderly care center in Køge which can be seen in a video.^14^

### 5.3 Beverage Delivery

It is important for people to keep hydrated during the day. Lack of hydration can lead to health issues, especially among the elderly. The SMOOTH-robot can help to keep people hydrated in the beverage delivery scenario described below. To be able to serve beverages, the SMOOTH-robot needs a tray for holding cups. Such an add-on was designed by the company Robotize, keeping attention on low weight and avoiding spillage. The tray (see Figure 3A) can hold up to 30 cups at a time. The cup holes are triangulated circles, to ensure a tight fit and avoid wobbling. For the robot to detect when a cup is removed, we opted for mounting infrared distance sensors on the bottom of the tray which allow easy detection of the cups’ presence (see Figure 3B). This information is important as the robot needs to end its drink-offering dialogue when a drink has already been taken.

Due to the Corona situation, we were not allowed to test the use case in elderly care centers anymore, and thus, we needed to find another facility. Fortunately, the beverage-serving use case is easily transferable to other scenarios. We chose a meeting area at the University of Southern Denmark where people also eat lunch (see Figure 3C).

The robot is best equipped to handle reception type scenarios where people are standing up, as the people detection algorithm provides the most precise results in this case, but it can also handle people sitting at tables, such as when they are having lunch at a cafeteria. We foresee two distinct situations which we have addressed separately: a) when there are not too many people in a given area, such that the robot has much space for navigation, and b) when people are positioned more densely, such that approach positions cannot be chosen with a certain degree of freedom. In the first scenario (see Figure 3C), the robot is able to observe and group people before making decisions on how to approach them, as can be seen in a video.^15^ In the second scenario, when the robot is not able to navigate easily between groups of people due to lack of space, the robot was equipped to act spontaneously without much prior planning.

The first scenario is shown in Figure 3C. Here, the robot starts by scanning its surroundings and estimating the pose of all detected people. Next, it assigns people to different groups according to the procedure described in Section 4.3. After grouping, the robot selects an approach point which would make it visible to most group members (see Figure 5C). It then uses the socially aware navigation described in Section 4.3 to reach the specific approach point. Once it arrives at this location, it switches to the interaction mode, where it scans for people’s visual attention as determined by their gaze direction using the appearance-based gaze detection algorithm (see Figure 9B). The robot establishes a mutual gaze with the person looking at it, using its simulated eyes on the front display of the head (see Figure 3A). Once eye contact is achieved, the SMOOTH-robot greets the person using speech communication (e.g., “Sorry to disturb you, but … ” or “Good evening”). Our analysis shows that our dialogue initiations are 100% successful, and as many as 78% of all people addressed actually respond verbally to the robot. The robot then offers the user something to drink, for example, “can I offer you some water?” Already, 59.4% accept the robot’s offer at this point. If people reject the robot’s offer, the robot can try to convince them with utterances conveying the importance of hydration, jokes, and persuasive messages, like “most women actually do take something to drink” [see Section 4.4 and Fischer et al. (2020a); Palinko et al. (2020)].

In the second scenario where no planning is possible due to the density of people in the area, the SMOOTH-robot moves around in a pseudo-random fashion and switches to the interaction mode as soon as it notices any person in its camera view (Naik et al., 2021). In this case, special attention needs to be paid not to address the same people multiple times. This is achieved by memorizing the physical location of the person whom the robot interacted with. As this assumes static people, it could be improved by using face recognition algorithms in the future. Once a person is detected in the robot’s field of view, the SMOOTH-robot stops and gazes toward them. Then it turns its body in the same direction, while the eyes are keeping the proper eye contact, simulating the vesibulo-ocular reflex. While turning, the robot greets the person and uses information about their gaze to determine if a person is interested in interacting with it. If it detects that person to be looking at it, it starts to offer water (“Excuse me, would you like a bottle of water?”). Once a bottle is removed from the tray or if a timeout is reached, the robot continues on its pseudo-random path to find more people to interact with.

During initial studies of beverage serving, gaze was found to be a very useful tool for facilitating the interaction between people and the robot. In one of the studies, we found that when approaching groups of people, the person gazed at was most often the one responding to the robot’s initiatives, unless that group was dominated by a particular person who was especially eager to talk to the robot Palinko et al. (2020). In the same experiment, we found that gazing at the person fortified the robot’s verbal communication, which resulted in people lifting their glasses more often when there was mutual gaze between them Fischer et al. (2020a). Table 1 shows the mean values and standard deviations of people lifting their cups, saying “skål” (“cheers” in Danish), and drinking water.

**TABLE 1:** Means (and standard deviations) of participants drinking, lifting their glass, and saying “skål” in response to the robot’s utterance (Fischer et al, 2020a).

	N	Drinks	Lifts	Says skål
No gaze	22	0.36 (0.49)	0.18 (0.39)	0.32 (0.48)
Gaze	20	0.55 (0.48)	0.65 (0.49)	0.70 (0.47)

## 6 Discussion

In this article, we introduced a novel assistive robot and a couple of functionalities that are realized on the developed platform. We made careful design choices concerning the technical complexity, the degree of anthropomorphism, the price it can be produced for, and its flexibility to be applied in various contexts, for which a modular design was decisive. We demonstrated the robot’s potential by means of three use cases and also described a wider range of possible applications.

We also reflected on the difficulty of entering the market with these kinds of robots, pointing to a number of obstacles former attempts have been facing. Our robot aims at filling a gap between logistic robots that are now widely used in companies and large institutions and over-complex robots that lack stability and affordable cost models.

For going from our prototype at TRL 5–6—where we are now in the development—to a successful product, it will be important to balance the complexity of the robot behaviors with what is technically achievable using state-of-the-art perception and control modules. Here, stability of behaviors is to be favored compared to sophisticated but unrealistic human–robot interaction schemes. Furthermore, the limits of the state of the art that still hinder the realization of smooth interactions should be taken into account. In addition, the choice of good market entry points will be crucial and so will affordable running costs. For that, not only the price of the robot will be decisive but also low idle times that can be achieved by using different modules for different purposes as shown in Figure 2.

## Data Availability

Since the project has ended in January 2021, in order to comply with GDPR regulations, most raw data was deleted. The remaining data supporting the conclusions of this article will be made available by the authors without undue reservation.

## Footnotes

1
www.mobile-industrial-robots.com

2
www.aethon.com

3 SMOOTH (2017–2021): Seamless huMan-robot interactiOn fOr THe support of elderly people, smooth-robot.dk

4
www.parorobots.com

5
www.care-o-bot.de

6
www.mojin-robotics.de

7
www.ropha-projekt.de

8
www.jibo.com

9 Workshop 13: Exploring Responsible Robotics Hands-On: A Conference Lab on Three Use Cases (SMOOTH Project)) Organizers: Kerstin Fischer, University of Southern Denmark, Denmark, Johanna Seibt, Aarhus University, Denmark, Norbert Krüger, University of Southern Denmark, Denmark. Robophilosophy 2018—Envisioning Robots In Society: Politics, Power, And Public Space, February 14–17, 2018 @University of Vienna, Austria

10
www.robotize.com

11
https://github.com/dialogos-project/dialogos

12
www.manoonpong.com/Smooth/D42/Smooth_UC2_Learning.mp4

13
https://nextcloud.sdu.dk/index.php/s/Bc6D7w6aFomfB3p

14
www.manoonpong.com/Smooth/M30/testing_koge/kogeII_20190912_poulGuiding_1_blur.mp4

15
https://www.youtube.com/watch?v=423Lg6LhsLM
